# Identification of Guanosine 5′-diphosphate as Potential Iron Mobilizer: Preventing the Hepcidin-Ferroportin Interaction and Modulating the Interleukin-6/Stat-3 Pathway

**DOI:** 10.1038/srep40097

**Published:** 2017-01-05

**Authors:** Stanzin Angmo, Neha Tripathi, Sheenu Abbat, Shailesh Sharma, Shelley Sardul Singh, Avishek Halder, Kamalendra Yadav, Geeta Shukla, Rajat Sandhir, Vikas Rishi, Prasad V. Bharatam, Hariom Yadav, Nitin Kumar Singhal

**Affiliations:** 1 National Agri-food Biotechnology Institute (NABI), S.A.S. Nagar, Punjab, India; 2 National Institute of Pharmaceutical Education and Research (NIPER), Sector-67, S.A.S. Nagar, Punjab, India; 3 Department of Biochemistry, Panjab University, Chandigarh, India; 4 Department of Microbiology, Panjab University, Chandigarh, Punjab, India; 5 National Institutes of Health (NIH), 9000 Rockville Pike, Bethesda, Maryland, USA

## Abstract

Hepcidin, a peptide hormone, is a key regulator in mammalian iron homeostasis. Increased level of hepcidin due to inflammatory conditions stimulates the ferroportin (FPN) transporter internalization, impairing the iron absorption; clinically manifested as anemia of inflammation (AI). Inhibiting hepcidin-mediated FPN degradation is proposed as an important strategy to combat AI. A systematic approach involving *in silico, in vitro, ex vivo* and *in vivo* studies is employed to identify hepcidin-binding agents. The virtual screening of 68,752 natural compounds *via* molecular docking resulted into identification of guanosine 5′-diphosphate (GDP) as a promising hepcidin-binding agent. The molecular dynamics simulations helped to identify the important hepcidin residues involved in stabilization of hepcidin-GDP complex. The results gave a preliminary indication that GDP may possibly inhibit the hepcidin-FPN interactions. The *in vitro* studies revealed that GDP caused FPN stabilization (FPN-GFP cell lines) and increased the FPN-mediated cellular iron efflux (HepG2 and Caco-2 cells). Interestingly, the co-administration of GDP and ferrous sulphate (FeSO_4_) ameliorated the turpentine-induced AI in mice (indicated by increased haemoglobin level, serum iron, FPN expression and decreased ferritin level). These results suggest that GDP a promising natural small-molecule inhibitor that targets Hepcidin-FPN complex may be incorporated with iron supplement regimens to ameliorate AI.

Anemia of inflammation (AI) is one of the most common manifestations of iron deficiency in the patients with inflammatory conditions^1^,^2^,^3^. AI is responsible for hypoferremia, with consequent iron-restricted erythropoiesis. Various studies have reported that hepcidin, a 25 amino acid cysteine-rich cationic peptide hormone, secreted from liver, is the key causative factor in AI^4^,^5^. Hepcidin synthesis and subsequent secretion is regulated by inflammation, hypoxia, erythropoiesis and iron stores within macrophages. The inflammation causes iron sequestration in macrophages, resulting into an excessive hepcidin production^2^,^6^,^7^. Ferroportin (FPN), a cellular iron transporter, is mainly expressed at the surface of hepatocytes, macrophages and enterocytes. Hepcidin binding to FPN triggers ubiquitination, endocytosis and degradation of FPN, which subsequently leads to reduced iron absorption^7^,^8^,^9^,^10^.

Hepcidin production from hepatocytes is regulated by multiple signalling pathways such as BMP-SMAD pathway and IL-6 *via* JAK STAT3 pathway^11^. Role of hepcidin agonists and antagonists in treatment of AI has been already established^12^. The currently employed strategies to prevent hepcidin-mediated FPN degradation include inhibiting hepcidin expression^13^,^14^,^15^, using anti-hepcidin agents^16^,^17^ and FPN binding agents^18^. The specifically employed therapeutic agents include anti-hepcidin antibodies^19^, BMP inhibitors (e.g. dorsomorphin)^20^, antagonists of BMP signaling (e.g. soluble hemojuvelin)^13^ and inhibition of SMAD signaling (e.g. glycol-split non-anticoagulant heparins)^17^. Anti-hepcidin Spiegelmer NOX-H94 a biostatic aptamer was reported to prevent hepcidin-induced FPN degradation^21^. LY2928057, a humanized IgG4 monoclonal antibody is a high affinity FPN binding agent that inhibits the hepcidin-FPN binding^16^. *Angelica sinensis* polysaccharide (ASP) was reported to suppress the expression of hepcidin in rats with AI^14^. Tocilizumab a monoclonal antibody was identified as an effective inhibitor of hepcidin production^22^. Fursultiamine is identified as a hepcidin antagonist that blocks the interaction of hepcidin-FPN disulfide bond^18^. Recently, anti-hemojuvelin antibodies are reported to ameliorate anemia due to their hepcidin suppressive potential^23^. These approaches are limited by unclear metabolic profile, complex delivery mechanism, various side effects such as thrombocytopenia, elevated levels of aminotransferases and poor pharmacokinetic profile.

In this work, using virtual screening, molecular docking and molecular dynamics studies, a natural compound guanosine-5′-diphosphate (GDP; ZINC Database ID: ZINC08215481) was identified that shows sufficiently good binding affinity with hepcidin. Further *in vitro* and *in vivo* studies confirmed the role of GDP in preventing hepcidin-mediated FPN degradation, reversing iron restrictive effect of inflammation with increase in haemoglobin level. GDP has been established as a promising candidate for inhibiting hepcidin-FPN interaction, thus promoting an effective iron-mediated erythropoiesis.

## Results

### Molecular docking based identification of the putative hepcidin inhibitors

The 3D structure of hepcidin-25 was used to screen the libraries of natural compounds and identify the possible hepcidin-binding agents, which may be further evaluated using *in vitro* and *in vivo* studies. The initial molecular docking-based virtual screening of 68,752 natural compounds (obtained from ZINC database, Table S1) led to the identification of 12 molecules (Figure S1) as putative hepcidin-binding agents. A common structural feature in all these ligands is the presence of a negatively charged moiety (Figure S1), which favours the peptide-ligand interaction, due to positive surface potential of the peptide (Figure S2A). The molecular docking analysis revealed that the identified compounds interact in the cavity formed due to hairpin-like structure of peptide (Figure S2B). The molecular docking scores obtained from DOCK6 (Table S2) did not reveal much difference in the binding affinities of various ligands and therefore, extensive molecular docking studies were undertaken using GLIDE software. The glide docking score, emodel score and interacting residues for these selected ligands are shown in Table 1. Commonly interacting residues identified from molecular docking analysis for all the ligands were Phe9, Cys10, Cys13, Cys14, His15, Arg16, Ser17, Cys19, Gly20, Met21 and Cys22.

### Molecular dynamics simulations of hepcidin-GDP complex

**Figure 1:**
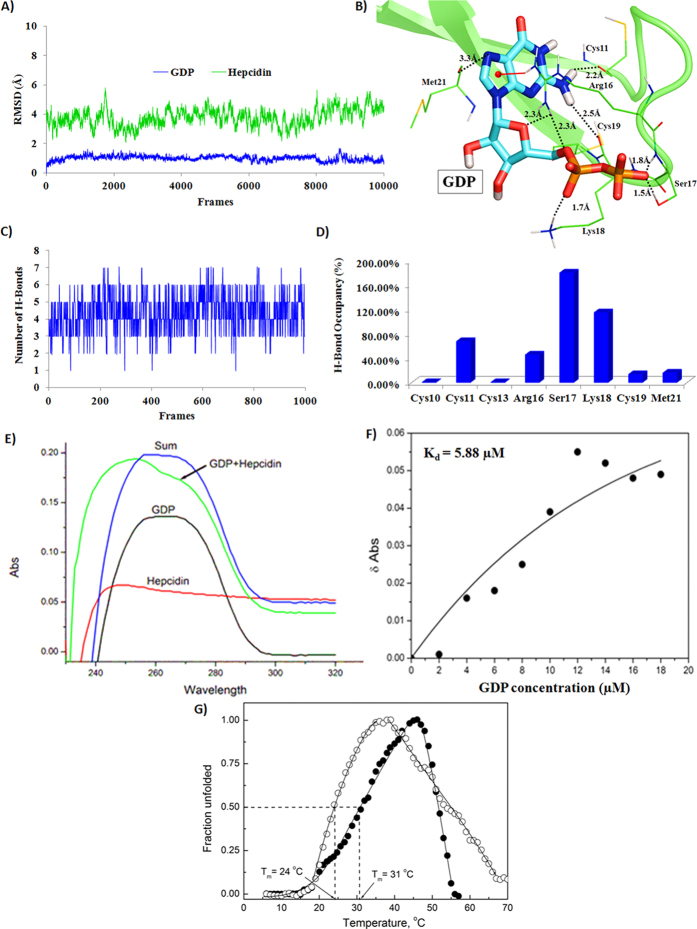
Molecular modeling and spectroscopic structural analysis of hepcidin-GDP complex. (**A**) RMSD of hepcidin (green) and GDP (blue) over 20 ns (500 frames/ns) simulation run; (**B**) Molecular recognition interactions of GDP with hepcidin; (**C**) Number of hydrogen bonds between GDP and hepcidin, over last 2 ns trajectory; (**D**) Hydrogen bond occupancies for residues over last 2 ns trajectory; (**E**) UV absorption spectra of GDP + hepcidin complex (green) indicates a complex formation of GDP and hepcidin (λ_max_ at 250 nm); (**F**) Delta absorbance as a function of GDP concentration. The K_d_ value was calculated to be 5.88 μM; (**G**) Thermal shift assay showed increased thermal stability of hepcidin peptide in the presence of GDP. T_m_ of hepcidin in the absence of GDP was 24 °C, which increased to 31 °C in the presence of equimolar GDP.

From the molecular docking analysis, ZINC08215481 (GDP) was found to be the top-scoring molecule (glide emodel score: −81.42 and glide docking score: −6.32). To get detailed insight into the stability of intermolecular interactions in hepcidin-GDP complex under dynamical conditions and to estimate the binding energy of GDP with hepcidin, molecular dynamics simulations (20 ns) were performed on the top ranking GDP conformation in the active site of hepcidin. The hepcidin-GDP complex was satisfactorily stabilized during the course of molecular dynamics simulations, as indicated by the Root Mean Square Deviation (RMSD) of hepcidin and GDP (Fig. 1A). The hepcidin RMSD graph (Fig. 1A, blue) clearly indicates that after 5 ns of simulation run the structure was stabilized. The RMSD fluctuation in case of GDP (Fig. 1A, green) is only about 0.5 Å throughout the simulation period. B-factor analysis (Figure S3A) and atomic fluctuation analysis (Figure S3B) indicated that the flexible region in the peptide corresponds to the ligand-binding region *i.e*. residues 11–21. The mobility of this region is consistent with the necessity of this region to undergo conformational changes upon inhibitor binding.

The binding energy along with the contributions from various energy components (van der Waals, electrostatic, polar solvation and nonpolar solvation interaction energies), calculated (using MMPBSA) over the last 2 ns trajectory is shown in Table 2. The binding energy of GDP for hepcidin was calculated to be −40.30 ± 4.71 kcal/mol. The electrostatic energy component of GDP is the predominant contributing component to the overall binding energy. The reason behind more favourable electrostatic contribution in this case is presence of negatively charged phosphate group in the GDP and its interaction with the positively charged residues of hepcidin. Hence, electrostatic energy contribution is the main driving force behind the stabilization of hepcidin-GDP complex.

The detailed analysis of molecular interactions helped to identify the important molecular recognition centres for hepcidin-binding agents. The analysis of hydrogen bond occupancies for hepcidin residues involved in interaction with GDP was performed over the last 2 ns (1000 frames) trajectory. On an average, GDP showed six stable hydrogen bonds with hepcidin (Fig. 1C). The prominent hydrogen bonds were formed by Cys11, Arg16, Ser17 and Lys18, as indicated by high occupancy values (Fig. 1D). Other residues which also showed intermittent hydrogen bonds are Cys10, Cys13, Cys19 and Met21. The per residue decomposition energy analysis indicated a high energy contribution of these residues for GDP binding (Figure S4). Thus, the *in silico* studies indicate that GDP may be a suitable candidate as a hepcidin-binding molecule and therefore, was taken up for further evaluation.

### GDP forms a complex with hepcidin

The UV-visible absorption spectra analysis (Fig. 1E) showed that GDP exhibits a λ_max_ at 260 nm, whereas hepcidin showed a λ_max_ at 243 nm. The sum of the two absorption spectra (for GDP and hepcidin) has a λ_max_ at around 260 nm. The GDP + hepcidin solution has a λ_max_ at 250 nm (hypsochromic shift). The results indicate complexation of hepcidin with GDP. The dissociation constant (K_d_) for hepcidin-GDP complex was calculated to be 5.88 μM (Fig. 1F), indicating towards a favourable complexation for GDP and hepcidin. The thermal denaturation analysis of hepcidin was performed to calculate the melting temperature (T_m_) in the presence and absence of GDP. T_m_ is the temperature at which fraction of folded hepcidin is equal to unfolded hepcidin. Hepcidin T_m_ was increased from 24 °C to 31 °C in presence of equimolar GDP (Fig. 1G). This suggests that GDP binds to the native form of peptide, thus corroborating the molecular modeling studies.

### GDP inhibits hepcidin-induced FPN internalization in FPN-GFP cell lines

**Figure 2:**
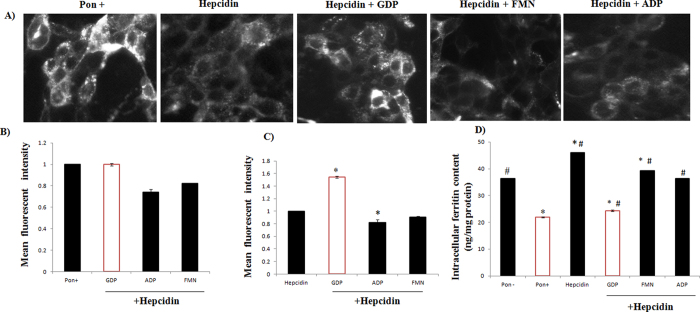
Effect of compounds on hepcidin mediated ferroportin degradation with cellular iron export. (**A**) FPN-GFP images of compounds representing GDP as potent hepcidin inhibitor with FPN retention on cellular surface in comparison with other organic phosphates. The Ponasterone (Pon^+^) induced cells were treated with hepcidin. Various compounds under evaluation were added to Pon^+^ induced cells with hepcidin. The baseline correction was employed using blank (Pon^−^). (**B**,**C**) The effect of GFP-FPN fluorescence intensity on GDP and other compounds was quantified using flow cytometer. P values were calculated using Holm-Sidak method.‘*’with P ≤ 0.05. (**D**) GDP and Pon^+^ prevented hepcidin-induced FPN internalization with decrease cellular iron efflux as compared to hepcidin, Pon^−^ and other compounds. P values were calculated using one-way ANOVA. ‘*’with P ≤ 0.05 *vs*. Pon^−^, ‘#’with P ≤ 0.05 *vs*. Pon^+^.

*In silico* and biophysical analysis suggest that hepcidin and GDP forms a stable complex. The inhibitory potential of GDP in hepcidin-FPN complexation was speculated using HEK293T cell line that expresses an inducible construct of FPN and C-terminal Green Fluorescent Protein (FPN-GFP)^24^, under the control of the ecdysone-inducible promoter. The ecdysone analog, ponasterone A, is used to induce expression of FPN-GFP. Cells treated with inducer Ponasterone (increase fluorescence) were used as the positive control and hepcidin-treated cells (decrease fluorescence) as the negative control. The epifluorescence microscopic analysis clearly showed that addition of hepcidin to FPN-GFP cell lines reduces the fluorescence intensity. The GDP + hepcidin treatment reversed this effect with cell-surface retention of GFP-FPN fluorescence intensity (Fig. 2A). Specificity of GDP in inhibiting FPN-Hepcidin complex was demonstrated using organic phosphate FMN and ADP that led to reduced cell surface retention of fluorescence signals in FPN-GFP expressing cell line. To further validate the hepcidin inhibitory potential of GDP on FPN-GFP complex, flow cytometry was used (Fig. 2B,C). Relative mean fluorescence fold change indicated that GDP treatment sustained more FPN-GFP fluorescence than control (Pon^+^, hepcidin treated cells) suggesting that GDP-hepcidin interaction significantly prevented the hepcidin-mediated FPN internalization, resulting into decreased intracellular ferritin level as compared to control (Pon^−^, Hepcidin) (Fig. 2D). Other organic phosphates (FMN and ADP) failed to interact with hepcidin and thus, proved ineffective in preventing hepcidin-mediated FPN degradation.

### GDP increases FPN mediated cellular iron efflux

**Figure 3:**
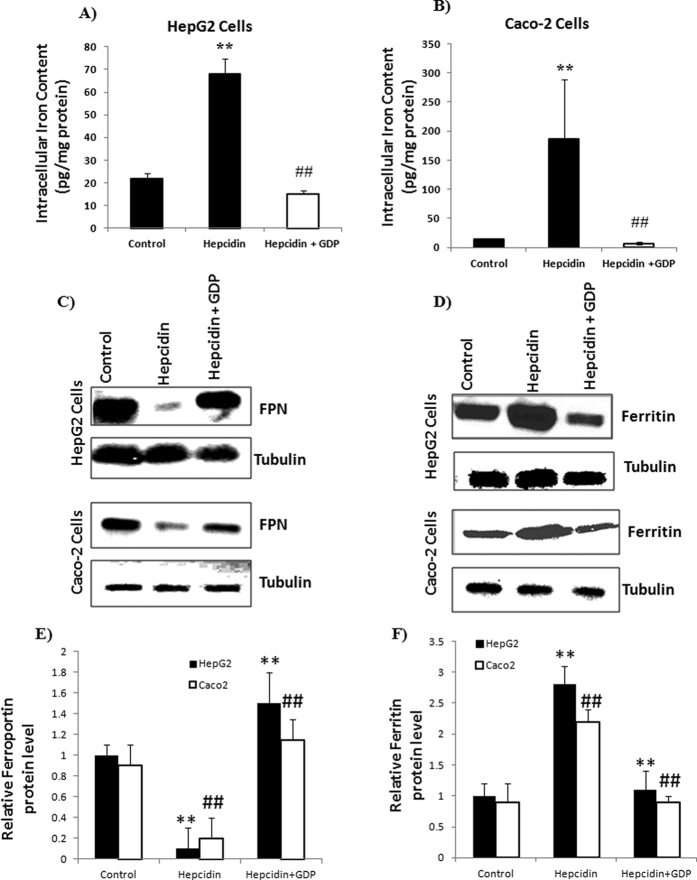
Protein expression and intracellular iron content level in HepG2 and Caco-2 cells. (**A**,**B**) Intracellular iron content was increased due to hepcidin treatment, whereas GDP treatment reversed this effect with decreased intracellular iron content level. (**C**,**D**) GDP prevented hepcidin-mediated FPN internalization with decrease iron storage ferritin level. (**E**,**F**) Immunoblot were scanned and densitometry was used to quantify the level of ferritin and FPN proteins relative to tubulin densities. Data were normalized to mRNA expression of a housekeeping gene, *GAPDH*. P values were calculated using one-way ANOVA. ‘**’ with P ≤ 0.01 control vs hepcidin and ‘**’ with P ≤ 0.01 hepcidin vs hepcidin + GDP in HepG2 cells. ‘^##^’ with P ≤ 0.01 control vs hepcidin and ‘^##^’ with P ≤ 0.01 hepcidin vs hepcidin + GDP in Caco-2 cells.

The stable binding of GDP with hepcidin (illustrated using *in silico* and UV-vis spectrophotometry) prevented hepcidin-induced FPN internalization (*in vitro*). Treatment with MANT-GDP (10 μM, a fluorescent analogue of GDP), respective fluorescence signals were observed in the HepG2 and Caco-2 cells confirming internalization of GDP within the cells (Figure S5). To prove this hypothesis in relevance to iron absorption, intracellular iron and iron storage ferritin protein levels were evaluated in Caco-2 and HepG2 cells. Hepcidin (1 μM) addition dramatically increased the intracellular iron concentration due to cellular iron retention, whereas addition of GDP (10 μM) reversed this effect by inhibiting the action of hepcidin (Fig. 3A,B). In parallel, protein expression of FPN and ferritin showed GDP was effective in suppressing hepcidin action with increased FPN expression and reduced iron storage ferritin level (Fig. 3C,D). GDP treatment significantly increased FPN protein level whereas, ferritin protein levels were significantly decreased compared to control in HepG2 and Caco-2 cells. Thus, GDP prevents hepcidin-induced FPN internalization with effective cellular iron efflux (Fig. 3E,F).

### GDP administration with iron supplement increases iron absorption in normal mice

**Figure 4:**
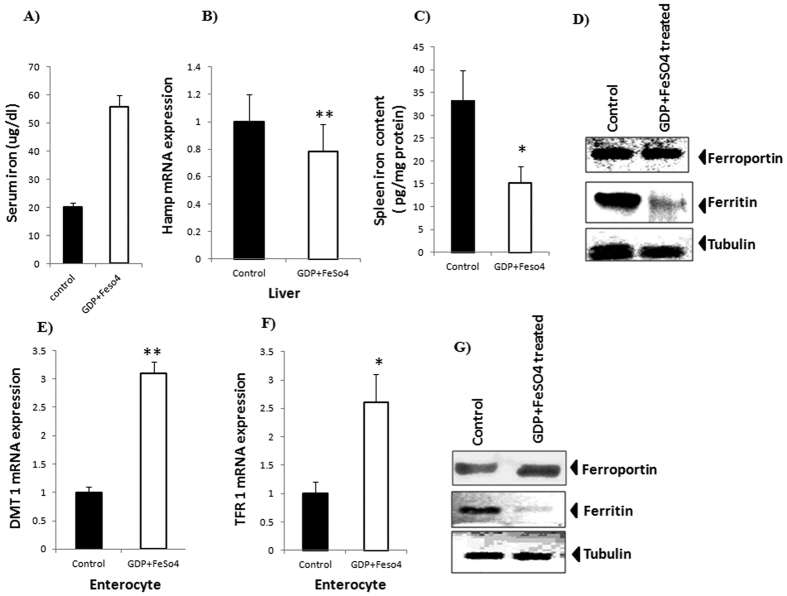
Effect of GDP + FeSO_4_ in response to normal body iron homeostasis. (**A**) GDP + FeSO_4_ significantly increase the serum iron concentration. (**B**) Decrease in Hamp expression is observed with treatment with GDP + FeSO_4_ in liver. GDP + FeSO_4_ in liver. (**C**,**D**) Decreases in spleen iron content level were observed along with increase FPN and decrease iron storage ferritin level. (**E**,**F**) Relative increase in mRNA expression of Divalent metal transporter 1 (DMT1) and Transferrin receptor 1 (TFR1) were observed in enterocyte. (**G**) Protein expression in enterocyte revealed increase FPN expression with reduced ferritin level for effective cellular iron efflux. Tubulin is used as an internal control. Results are normalized to *GAPDH* and expressed as mean ± SD for *n* animals (n = 8/group). P values were calculated using student *t* test. **P ≤ 0.01 *P ≤ 0.05.

The *in silico* and *in vitro* studies provided a clear evidence for GDP-hepcidin interaction that prevents hepcidin-induced FPN internalization. To establish the physiological and biological importance of GDP treatment on whole body iron homeostasis, one dose response study was carried out in normal wild type mice. Serum iron level was found significantly higher after GDP treatment, relative to control mice (Fig. 4A). Experimental mice received GDP along with FeSO_4_ intraperitoneally and decrease in *Hamp* mRNA was observed relative to control mice (Fig. 4B). However, spleen iron content was decreased by 46% in GDP treated mice, compared to control mice (Fig. 4C). Protein expression data demonstrate increased FPN expression with decreased iron storage ferritin level in spleen for effective cellular-mediated iron export (Fig. 4D). Interestingly, GDP + FeSO_4_ in enterocyte showed increased mRNA expression of DMT1 and TFR1 indicating effective iron distribution (Fig. 4E,F). This was associated with elevated protein level of iron exporter FPN with decreased ferritin level in enterocyte (Fig. 4G). The results suggested that GDP + FeSO_4_ can suppress hepcidin action in normal body iron homeostasis that potently reduced the iron storage ferritin level, thus preventing hepcidin-induced internalization of FPN with increased iron absorption.

### GDP ameliorates turpentine-induced AI in mice

**Figure 5:**
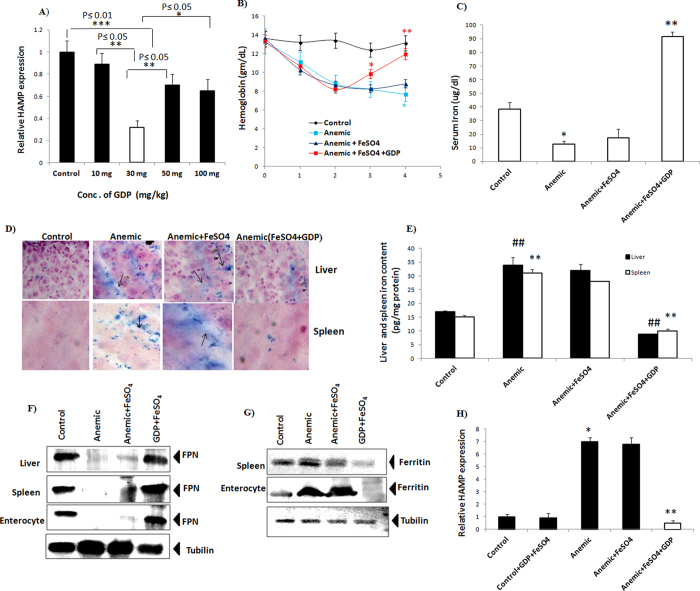
Turpentine induced AI ameliorated with GDP + FeSO_4_ treatment. (**A**) Dose response study at different concentration of GDP in response to *Hamp* gene expression in liver (**B**) GDP + FeSO_4_ corrected anemia induced with turpentine with significant increase in haemoglobin level. (**C**) Increase in serum iron is exhibited on treatment with GDP + FeSO_4_. (**D**,**E**) Tissue specific iron distribution in anemic and anemic + FeSO_4_ showed increase iron deposits due to hepcidin-induced FPN internalization whereas, GDP + FeSO_4_ reversed this effect with decrease in iron accumulation paralleled with decrease liver and spleen iron content level. ‘^##^’ with P ≤ 0.01 control vs anemic and ‘^##^’ with P ≤ 0.01 anemic + GDP + FeSO_4_ vs anemic in liver. ‘**’ with P ≤ 0.01 control vs anemic and ‘**’ with P ≤ 0.01 anemic + GDP + FeSO_4_ vs anemic in spleen. (**F**) Protein expression analysis revealed increase FPN expression in liver, spleen and enterocyte with effective cellular mediated iron efflux. (**G**) Decrease in iron storage ferritin level were observed in spleen and enterocyte thus, improving hypoferrmia. (**H**) Gene expression analysis showed decrease hepatic *Hamp* mRNA expression in comparison to Control + GDP + FeSO_4_. Results are normalized to *GAPDH* and expressed relative to controls. *n* = 8/group. P values were calculated using One-way ANOVA and Two-way ANOVA. ‘*’ with P ≤ 0.05 control vs anemic ‘**’ with P ≤ 0.01 anemic + GDP + FeSO_4_ vs anemic.

Dose-response study was carried out in mice at different GDP concentrations to calculate the optimal concentration in response to *Hamp* mRNA expression in liver (Fig. 5A). Dose-response study revealed that GDP concentration of 30 mg/kg was the most effective in suppressing *Hamp* mRNA expression level. To examine the preclinical relevance of GDP in treatment of AI, BALB/c mice were injected once a week with turpentine for 2 week to develop AI. Next, AI mice were treated with GDP + FeSO_4_ intraperitoneally every 24 hours for 2 week. Treatment with GDP + FeSO_4_ significantly increased haemoglobin level as compared to anemic and anemic + FeSO_4_ mice (Fig. 5B). Additionally, significant increase in serum iron level was observed in GDP + FeSO_4_ treated mice (Fig. 5C). Tissue specific iron deposits were clearly observed in the anemic and anemic + FeSO_4_ treated tissues (liver and spleen) indicating hepcidin- mediated FPN internalization with increased iron accumulation, whereas addition of GDP + FeSO_4_ reversed this effect possibly *via* stabilization of FPN with decreased liver and spleen iron level (Fig. 5D,E). This decrease was paralleled with increased FPN level (liver, Spleen, and enterocyte) (Fig. 5F) and reduced iron storage ferritin level (spleen and enterocyte) (Fig. 5G) for effective iron-mediated erythropoiesis thus improving hypoferremia. These results corroborate well with the proposed hepcidin-GDP complex as a valid molecular target to prevent internalization of FPN resulting in avaibility of iron for haemoglobin synthesis. Increased hepatic *Hamp* expression were observed in anemic and anemic + FeSO_4_ group but addition of GDP + FeSO_4_ significantly decreased *Hamp* mRNA expression as compared to anemic and control + GDP + FeSO_4_ group (Fig. 5H). Interestingly, additional decrease in the hepcidin expression at transcriptional level was noticed due to treatment with GDP + FeSO_4_. Lipopolysaccharide (LPS) induced inflammation increased IL6-mediated phosphorylation of Stat-3, whereas treatment with GDP + FeSO_4_ reduced Stat-3 phosphorylation in mice hepatocyte resulting in decreased hepcidin expression (Figure S6).

## Discussion

AI is a normocytic anemia, common among patients with chronic infection and inflammatory disorders^1^. The increased hepcidin level is identified as the leading cause of AI. The hepcidin-FPN complexation results into the ubiquitination of FPN, its subsequent internalization into the cell and finally, lysosomal degradation^24^,^25^. The currently employed strategies mainly focus on regulating the hepcidin expression and targeting FPN. Some of the synthetic drugs such as fursultamine^18^, thioxolone^18^ and chloroquine^24^ are reported to prevent the hepcidin-induced FPN internalization, with retention of FPN on cellular surface and subsequently, the cellular iron efflux. The LDN193189^10^, heparin^17^ and HJV. Fc^26^ inhibit the hepcidin expression by binding to BMPs and blocking BMP-SMAD signalling pathway. The mechanism of action for these drugs is not mediated via hepcidin binding. Lexapteptid pegol^27^ (RNA aptamer) and 12B9m^28^ (monoclonal antibody) are reported to bind hepcidin and inhibit its activity. The basic disadvantage exhibited by these reagents is their processing and delivery.

In this study, we adopted a systematic strategy for identification of natural compounds as hepcidin-binding agents. Recently, hepcidin antagonists were identified by screening various synthetic chemical libraries^18^. In this work, the natural compounds libraries are utilized to identify the possible hepcidin binding agents by employing the virtual screening, molecular docking and molecular dynamic analysis. The results indicated that GDP can favourably bind to hepcidin (binding energy of −40.30 ± 4.71 kcal/mol). The surface electrostatic potential of the receptor cavity site of hepcidin was revealed to be highly positive (Figure S2), leading to the favourable accommodation of GDP phosphate groups. This indicates towards the importance of electronegative functional groups in hepcidin-GDP interaction. The macromolecular and peptide residues, playing critical role in hepcidin-FPN interaction are reported in literature^29^,^30^. The hepcidin residues Phe9, His15, Arg16, Ser17, Lys18, Cys19, Gly20, Met21 and Cys22 were previously identified to be important for hepcidin-FPN interaction (Table S3). In the current study, hepcidin residues Cys11, Arg16, Ser17 and Lys18 were found to be important for hydrogen-bonding interaction with GDP (Fig. 1B). The per-residue decomposition energy analysis showed that Cys11, Arg16, Ser17, Lys18 and Cys22 (Figure S4) have a higher contribution to the stability of hepcidin-GDP complex. These results indicate that GDP structure can block the crucial residues of hepcidin, making it unavailable for interfacial contact with FPN, which can possibly increase iron absorption in the body. This physical binding of hepcidin and GDP was further established by UV-spectroscopic and thermal shift assays.

To validate the proposed hypothesis from *in silico* and experimental studies, initially GDP internalization into cells (HepG2 and Caco-2) was validated using MANT-GDP fluorescence signals (Figure S5). Further FPN-GFP cell line assay was performed to evaluate the effect of GDP on hepcidin-FPN interaction. The results demonstrated that the hepcidin treatment reduced the GFP-FPN fluorescence with distribution of FPN from cell surface to intracellular vesicles. Whereas, GDP binding to hepcidin prevented internalization of FPN, thereby retaining the cellular membrane localization of FPN with reduced appearance of FPN distribution in intracellular vesicles (Fig. 2). We found decrease in intracellular ferritin level in GDP treated cells compared to Pon^−^ and hepcidin, whereas other organic phosphate failed to show such effect. These *in vitro* assays on HepG2 and Caco-2 cell lines supported the protective effect of GDP on iron efflux transporter FPN, resulting into decrease iron storage ferritin level (Fig. 3).

Elevated hepcidin level in normal iron homeostasis and AI (inflammatory conditions) is the major cause of decreased liver and macrophage iron level, hence reducing FPN stability with iron sequestration leading to iron restrictive anemia. The normal physiological concentration of GDP (as per the literature report) in mouse liver ranges from 14 ± 3.5 to 21 ±  2 μmole/100 g tissue^31^. Based on our preliminary investigation, it was hypothesized that targeting hepcidin to inhibit its interaction with FPN can be an important strategy to overcome the iron-restricted erythropoiesis. Based on this hypothesis, first we investigated the GDP and iron supplement (FeSO_4_) effect on biological and physiological response in normal mice iron homeostasis. We found decrease in *Hamp* expression with increased serum iron and reduced spleen iron content. This decrease was associated with reduce ferritin level in spleen and enterocyte with effective cellular-mediated iron efflux through basolateral membrane (Fig. 4D–G). This provides clear evidence that GDP is active in normal physiological conditions and can act as a potent hepcidin inhibitor.

Our next objective was to evaluate the efficacy of GDP in overcoming the AI. The turpentine induced AI model is a well-established system to study the effect of inflammation on iron regulation and erythropoiesis in mice and humans^32^,^33^,^34^. Darpoepietin alfa, a novel erythropoiesis stimulating protein alleviates anemia in a dose-dependent manner in AI rodent model^35^. The current study established that in turpentine-induced anemic mice, the elevated hepcidin level restricts iron efflux, causing hypoferrmia. We used this model of AI to test the hypothesis that the inhibition of hepcidin would stabilize FPN transporter that would in turn promote iron mobilization for haemoglobin synthesis. GDP + FeSO_4_ treatment in anemic mice increased haemoglobin level compared to control mice, thus assists in correcting the anemic state. Serum iron level was significantly increased in GDP + FeSO_4_ treated anemic mice, possibly due to decrease in hepcidin concentration and increased FPN level at cellular membrane surface. This increase in serum iron was associated with decreased iron distribution in reservoir tissue (liver and spleen). This decrease was associated with increased protein expression of FPN in spleen, liver, and enterocyte, and decrease iron storage ferritin level in spleen and enterocyte thus improving the hypoferrmia by reversing the iron restrictive effect of inflammation. In present study FeSO_4_ was used as iron supplement regime in conjunction with GDP to address chronic anemic condition. Present results provide compelling evidence that binding of GDP inhibit hepcidin action. The results support the novel hypothesis that GDP along with iron supplement regime can inhibit the hepcidin-mediated FPN degradation that would promote release of cellular iron and will be an effective treatment for AI. Additionally, we found decreased *Hamp* level in mice suffering from AI after treatment with GDP + FeSO_4_ in comparison to anemic and control + GDP + FeSO_4_ (Fig. 5H). However, it can be concluded that GDP + FeSO_4_ may act at transcriptional as well as translational level. We have demonstrated the binding of GDP to hepcidin and simultaneous disruption of hepcidin-FPN complex. GDP + FeSO_4_ also decreases the *Hamp* mRNA level, pointing towards involvement of IL6/Stat-3 pathway where GDP is acting at transcriptional level. It regulates inflammatory response by decreasing phosphorylated Stat-3 that in turn decreases the expression of *Hamp* level (Figure S6 in supplementary information). Furthermore, we will be exploring the mechanism of GDP + FeSO_4_ action in inflammation (LPS) induced hepcidin expression *via* IL-6-inducible Stat-3 pathway.

In conclusion, this study illustrates the hepcidin binding potential of GDP using the molecular modeling techniques further validated by biochemical and biophysical method. The antagonist potential of GDP was hypothesised on the basis of its ability to block molecular recognition interactions of hepcidin-FPN complexation. This hypothesis was supported by *in vitro* and *in vivo* studies. These pharmacological interactions are accountable for maintaining the systemic body iron homeostasis and successful reversal of chronic anemia. Thus, it can be inferred that the GDP can be a robust candidate as a hepcidin binding agent, with higher preclinical and clinical relevance. The results obtained in this study pave the way for the design of hepcidin binding agents and overcome the inflammation-induced anemic conditions.

## Materials and Methodology

### Molecular docking based virtual screening and identification of hit molecules

The 3D structure of hepcidin-25 (PDB ID: 3H0T)^36^ was downloaded from Protein Data Bank. The structures of 68,752 natural compounds were obtained from ZINC database^37^ (Table S1). Molecular docking based virtual screening (using DOCK6)^38^ led to the selection of 12 ligands (Table S2), most of which were phosphate containing compounds.(Figure S1) To identify the best hit out of these 12 compounds, molecular docking studies were performed in Standard Precision (SP) mode using GLIDE 5.7 module^39^ of Maestro 9.3 package^40^. The 3D structures of ligands and peptide were prepared using LigPrep module and Protein Preparation Wizard module of Maestro9.3 package^40^. The ligand-receptor interaction grid was generated (inner box of 10 Å, outer box of 12 Å; peptide centroid: 14.31, −3.91, 9.55) using Receptor Grid Generation Wizard. The final pose selection was based on glide docking score, emodel score and molecular recognition interactions.

### Molecular dynamics simulations

To analyse the stability of hepcidin-GDP complex, MD simulations were performed using AMBER11 package^41^. The input files were prepared as per the standard protocol (see supporting information for details). After initial minimization of the system, gradual heating, density equilibration and 1 ns constant pressure equilibration were performed sequentially. Finally, production run for 20 ns was performed. The binding energy calculation for hepcidin-GDP complexation was performed over last 2 ns (1000 frames) trajectory using Molecular Mechanics-Poisson Boltzmann Surface Area (MM-PBSA) method^42^.

### Ultraviolet–visible spectra absorbance analysis

Ultraviolet–Visible (UV-Vis) spectroscopic titrations (Shimazdu 2700) were performed at room temperature using hepcidin (10 μM) and GDP (variable concentration; range: 0.5−1.25 μM; interval of 5 sec). The UV–Vis (220–320 nm) spectra were measured after 1 min interval and dissociation constants for hepcidin-GDP complexation were determined. Buffer baseline correction was employed for the UV spectra.

### Thermal-shift assay

Thermal shift assay was performed using Applied Bio systems 7500 Fast Real-Time PCR machine. Hepcidin peptide (28.64 μM) was heated in absence and presence of equimolar GDP from 6–70 °C at the rate of 1 °C/minute. Emission spectrum of bound *SYPRO orange* fluorescent dye (as peptide unfolds) was measured at 575 nm. Relative fluorescence signals were converted to fraction unfolded peptide and plotted as the function of temperature.

### FPN-GFP quantification using flow cytometry analysis

The FPN-GFP cell line (HEK293T) was a precious gift from Prof. Jerry Kaplan (University of Utah)^25^. The FPN-GFP cell line was maintained in a growth medium (DMEM with 2.0 g/L NaHCO_3_, 10% FBS, 400 μg/ml zeocin, 400 μg/ml G418, pH 7.2) supplied from Gibco (Thermo Fisher Scientific, USA) and 20 μM Ferric ammonium citrate^18^ (Sigma-Aldrich USA). The FPN-GFP cell line was induced for the GFP expression using 10 µM ponasterone (Sigma-Aldrich USA) and incubated for 18–20 h. After two washes with 1xDPBS, cells were treated with various combinations of hepcidin (Abcam USA) and organic phosphates *i.e*. GDP, adenosine diphosphate (ADP), riboflavin-5′-monophosphate (FMN). The experimental setup included cells induced with ponasterone (Pon^+^, positive control), cells induced with ponasterone and treated with 1 μg/ml hepcidin (negative control)^8^, and cells induced with ponasterone and treated with hepcidin, were further subjected to treatment with GDP (10 μM), ADP (10 μM) and FMN (10 μM), separately^18^. The background fluorescence was excluded by using ponasterone untreated cells (Pon^−^, blank). The intensity of green fluorescence was measured using flow cytometer BD LSFortessa (BD FACS Diva 8.0). Results were reported as fraction of the GFP intensity of untreated cells i.e. [F_X_–F_Pon_–F_untreated_/F_Pon_–F_untreated_], where F_pon_ is the fluorescence intensity of positive control; F_untreated_ is the fluorescence intensity in the blank; and F_X_ is the fluorescence intensity in the various samples.

### Ferritin measurement

Total cellular protein was extracted using Radio immunoprecipitation assay (RIPA) buffer (Sigma-Aldrich, USA) and a protease inhibitor cocktail (Sigma-Aldrich, USA). The ferritin level was determined using an enzyme-linked immunosorbent assay (ELISA)^43^, as per user manual. The results were normalized for the total protein concentration in each sample^18^. Protein concentration was determined using the bicinchoninic acid assay (BCA assay, Thermo Fisher Scientific, Invitrogen, USA).

### Cell line and cell culture

The Caco-2 and HepG2 cell lines (obtained from NCCS, Pune, India) were cultured at 37 °C in humidified air (95%) and 5% CO_2_ in Dulbecco Modified Eagle Medium (DMEM), supplemented with 1% (v/v) antibiotic solution and 10% FBS. The medium was changed every alternate day, and were passaged at approximately 85% confluence. Before treatment, cells were seeded in 6 well plates at an optimum density of 0.3 × 10^6 ^cells/well and confluent at 1.2 × 10^6 ^cells/well. The medium was removed and fresh DMEM was added before the experiment. Cells were treated with MANT-GDP (2′-(or-3′)-*O*-(*N*-Methylanthraniloyl) Guanosine 5′-Diphosphate (10 μM) (Thermo Fisher Scientific, Invitrogen, USA) a fluorescent analogue of GDP for 24 h. Images were acquired using (Leica inverted microscope). Each cell line was treated separately with 1 μM hepcidin^44^ (Abcam USA) and 10 μM GDP (Sigma-Aldrich, USA). The untreated cells were used as control.

### Quantitative gene expression studies

Total RNA was isolated from cell lines (Caco-2, HepG2) and from tissue sample (liver, enterocyte) using RNA extraction kit (Invitrogen, USA). The qualitative ratio metric analysis of RNA was done using Infinite 200 PRO Nano Quant (Tecan, Switzerland). RNA (2 μg) was reverse transcribed to single strand cDNA using cDNA synthesis kit (High capacity reverse transcription kit, Applied Bio system). Quantitative gene expression of *hamp*, TFR1 and DMT1 was quantified by qPCR (Applied Bio systems 7500 Fast Real-Time PCR machine), using gene specific primers (Table S4). Data was analysed using ΔΔC_t_ method (Biosciences, Qiagen, USA). Glyceraldehyde-3-phosphate dehydrogenase (GAPDH) was used as an internal control for normalization of quantitative gene expression data.

### Animal treatment

Male BALB/c mice (weighing 25 ± 3 g) were procured from the Central Animal House, Panjab University, Chandigarh, India. The animals were handled in accordance with the “Guidelines for the Care and Use of Experimental Animals” and were approved by the University Ethics Committee.

For short-term treatment experiment, BALB/c mice were treated with a single intraperitoneal injection of GDP + FeSO_4_ (GDP 30 mg/kg and FeSO_4_ 2 mg/kg) and control received FeSO_4_ only. Mice were killed after 24 h and tissues were isolated and stored at −80 °C for further studies.

For long-term experiment, AI condition was induced by injecting mice with turpentine oil (Sigma-Aldrich, USA. 0.1 ml/20 g of body weight)^33^,^45^ in intra-scapular fat for two weeks (3 injections/week). Animals were divided into four groups (n = 8/group) *i.e*. control (non-anemic mice), anemic group, anemic + FeSO_4_ group and anemic + FeSO_4_ + GDP group. To observe the effect of GDP on anemic state, the anemic + FeSO_4_ + GDP group were administered with FeSO_4_ (2 mg/kg) and GDP (30 mg/kg) intraperitoneally for next two weeks (3 doses/week). The haemoglobin level was measured every week. The mice were euthanized on the third week, to collect tissue samples. At the end of protocol, blood was collected by cardiac puncture, for measurement of serum iron and erythrocyte parameters. Dose response study was conducted at different GDP concentration in response to *Hamp* mRNA expression in liver. Tissues were isolated and were stored at −80 °C for further studies. Further liver and spleen tissue were isolated to measure iron level. To examine the effect of LPS on IL-6/Stat-3 pathway in inflammation induced mice model (See supporting information for details).

### Western blotting

Total cellular protein and tissue protein extract were prepared and western blotting was performed^10^,^15^. The extracted proteins were transferred to PVDF membranes (Invitrogen, USA) and incubated with primary antibodies *i.e*. anti-ferritin antibody (1:1000; Sigma-Aldrich, USA) anti-SLC-40A1 antibody (1:5000; Sigma-Aldrich, USA), anti phospho-Stat-3 (1:1000; Sigma-Aldrich, USA), Stat-3 (1:1000; Sigma-Aldrich, USA). Further the blot was incubated with secondary peroxidase-labelled antibodies, anti-rabbit IgG antibody (1:5000; Sigma-Aldrich USA) and anti-mouse IgG antibody (1:1000; Sigma-Aldrich USA) for 1 h. Anti-*α*-tubulin antibody was used as a loading control. PVDF membrane for immune-reactive band was detected by immunostaining kit (Sigma-Aldrich, USA). Immunoblot were quantified using ImageJ software, and band densities were normalized with the corresponding Tubilin band densities. The blot shown is representative of 3 independent experiments.

### Serum iron and intracellular iron measurement

Serum was separated immediately by centrifugation of the blood samples for 15 min at 4 °C. Serum, Caco-2 and HepG2 cells (approximately 0.3 × 10^6 ^cells/well) and tissue samples (liver and spleen) were used for acid digestion in the microwave accelerated reactor system (MARS6, CEM Corporation, USA). Intracellular iron concentration and iron contents of organs (liver and spleen) were estimated in the digested samples using inductively coupled plasma mass spectrometry (ICP-MS; 7700 × AgilentTechnologies, Santa Clara, CA).

### Tissue histology and hematologic parameter measurement

Tissue histology sections were obtained for each sample, fixed in 4% formaldehyde and embedded in paraffin. Thereafter, sections were stained with Perl’s Prussian blue to visualize iron deposits. Images were acquired using Leica Laser-Tech (GmbH, Heidelberg, Germany). Blood was obtained by retro-orbital phlebotomy of mice and collected in heparinized tubes. Erythrocyte parameters were determined using Drabkin’s reagent (Sigma-Aldrich, USA) using UV-vis spectrophotometer (λ_max_ at 540 nm).

### Statistical Analysis

Statistical differences between qRT-PCR data were analyzed using one-way and two-way analysis of variance (ANOVA) and Student’s t-test. Subsequently, Tukey’s multiple comparison procedure was employed to check the level of significance using Prism Graph Pad software (Graph Pad Software Inc, San Diego, CA, USA). FPN-GFP cell lines were quantified using flow cytometer. The results were analysed using Holm-Sidak method. The experiments were carried out in triplicate to calculate standard deviation (SD), standard error of mean (SEM) and level of significance (based on P value). In all the tests, *P* ≤ 0.05 and P ≤ 0.01 were taken as the criterion for statistical significance.

## Additional Information

**How to cite this article**: Angmo, S. *et al*. Identification of Guanosine 5′-diphosphate as Potential Iron Mobilizer: Preventing the Hepcidin-Ferroportin Interaction and Modulating the Interleukin-6/Stat-3 Pathway. *Sci. Rep.* **7**, 40097; doi: 10.1038/srep40097 (2017).

**Publisher's note:** Springer Nature remains neutral with regard to jurisdictional claims in published maps and institutional affiliations.

## Supplementary Material

Supplementary Information

## Figures and Tables

**Table 1:** Molecular docking results for the selected 12 compounds.

S. No.	Title	GLIDE emodel Score	Glide docking Score	Hydrogen bonds	Other residues within 5 Å	NH-π/π-π Interactions
1	**ZINC08215481**	−81.42	−6.32	His15, Arg16	Phe9, Cys10, Cys13, Cys14, Ser17, Cys19, Gly20, Met21, Cys22	Arg16
2	**ZINC04096694**	−68.55	−5.71	His15, Arg16, Lys18, Cys19, Met21	Ser17, Gly20	
3	**ZINC24433941**	−61.48	−4.47	Arg16, Met21	Cys14, His15, Met21	
4	**ZINC04654620**	−55.24	−4.04	His15, Arg16, Ser17, Met21	Phe9, Lys18, Cys19, Gly20, Met21	
5	**ZINC31169915**	−54.15	−4.48	His15, Arg16, Ser17, Lys18, Met21	Cys19, Gly20, Met21	Phe9
6	**ZINC04228295**	−54.10	−4.95	His15, Arg16, Met21	Ser17, Gly20	
7	**ZINC04095542**	−53.36	−4.72	His15, Arg16, Lys18, Cys19	Ser17, Gly20	
8	**ZINC31164026**	−50.47	−3.75	His15, Arg16, Lys18	Phe9, Ser17, Cys19, Gly20, Met21, Cys22	
9	**ZINC04221664**	−49.99	−3.58	His15, Arg16, Cys19, Met21	Phe9, Cys14, Ser17, Lys18, Gly20, Cys22	Arg16
10	**ZINC08551105**	−49.81	−4.19	His15, Arg16, Lys18	Ser17	Phe9, Cys19, Gly20, Met21
11	**ZINC13527007**	−48.48	−4.98	His15, Arg16	Ser17	
12	**ZINC13424733**	−40.74	−3.35	His15, Arg16, Lys18	Ser17	Phe9, Cys19, Gly20, Met21

The compounds are ranked on the basis of GLIDE emodel score. The top scoring ligand is guanosine 5′-diphosphate (ZINC08215481).

**Table 2:** Average binding energy for hepcidin-GDP complex (last 2 ns) along with its different energy components^a^.

Components	Energy (kcal/mol)
VDW	−28.17
EEL	−460.44
EPB	450.77
ECAVITY	−2.45
ΔG_gas_	−488.61
ΔG_solv_	448.31
ΔG_bind_	−40.30 ± 4.71

VDW, van der Waals energy as calculated by the MM force field; EEL, electrostatic energy as calculated by the MM force field; EPB, electrostatic contribution to the solvation free energy calculated by PBSA. ECAVITY, nonpolar contribution to the solvation free energy calculated by PBSA; ΔG_gas_, total gas phase energy *i.e*. sum of van der Waals and electrostatic energy from MM; ΔG_solv_, total solvation free energy *i.e*. sum of electrostatic and nonpolar contributions from PBSA. ΔG_bind_, final estimated binding energy calculated from these terms.
