# Supplementary material for: Identification of Guanosine 5′-diphosphate as Potential Iron Mobilizer: Preventing the Hepcidin-Ferroportin Interaction and Modulating the Interleukin-6/Stat-3 Pathway
Source: Sci Rep. 2017 Jan 5;7:40097. doi: 10.1038/srep40097 (PMC5214259; doi:10.1038/srep40097)
Supplement: Supplementary Information [file srep40097-s1.pdf]

Identification of Guanosine 5′-diphosphate as Potential Iron Mobilizer: Preventing the Hepcidin-Ferroportin Interaction and Modulating the Interleukin-6/Stat-3 Pathway

Stanzin Angmo,[1] Neha Tripathi,[2] Sheenu Abbat,[2] Shailesh Sharma,[1] Shelley Sardul Singh,[1] Avishek Halder[5], Kamalendra Yadav,[1] Geeta Shukla,[3] Rajat Sandhir[5], Vikas Rishi[1], Prasad V. Bharatam,[2] Hari Om Yadav,[4*] Nitin Kumar Singhal[1*]

[1]National Agri-food Biotechnology Institute (NABI), S.A.S. Nagar, Punjab, India;[2]National Institute of Pharmaceutical Education and Research (NIPER), Sector-67, S.A.S. Nagar, Punjab, India; [3]Department of Microbiology, Panjab University, Chandigarh, Punjab, India; [4]National Institutes of Health (NIH), 9000 Rockville Pike, Bethesda, Maryland, USA. [5]Department of Biochemistry, Panjab University, Chandigarh, India.

Running Title: GDP as an inhibitor of hepcidin action.

[*]**Corresponding Authors**:
Dr. Nitin Singhal PhD, Food science and Technology Department, National Agri-Food Biotechnology Institute, Mohali, Punjab, India.
nitin@nabi.res.in
Dr. Hari Om Yadav National Institutes of Health (NIH), 9000 Rockville Pike, Bethesda, Maryland, USA yadav@mail.nih.gov

Results

The detailed analysis of molecular interactions helped to identify the important molecular recognition centres for hepcidin binding agents. The analysis showed that the Arg16 forms a bifurcated hydrogen bond with the GDP α-phosphate group (N-H···O distance 2.3 Å) and ribose ring oxygen (N-H···O distance 2.3 Å). Further, NH···π interaction was observed between Arg16 and the purine moiety of GDP (Figure 1B). Several other hydrogen bonds such as Met21 and purine ring (3.3 Å), Cys19 and purine amino group (2.5 Å), Ser17 and β-phosphate group (1.5 Å and 1.8 Å), Lys18 and GDP α-phosphate group (1.7 Å), etc., were observed.

Methods

Molecular Dynamics Simulations

To analyse the stability of docked ligand GDP in the active site of hepcidin, MD simulations were performed using AMBER 11 package.[1] Restrained Electrostatic Potential (RESP) method[2] of *antechamber* module[3] was used for the partial atomic charge calculations. For the preparation of QC and macromolecules, General AMBER Force Field (GAFF)[4] and AMBER ff99SB force field were implemented, respectively. TIP3P water model[5,6] was used for the solvation of the hepcidin-GDP complex structure, creating a cubical solvent box, extended to 10 Å on each side of the complex. After initial minimization of the system, gradual heating was performed from 0 to 300 K under NVT ensemble. Subsequently, density equilibration was carried out under NPT ensemble followed by constant pressure equilibration for 1 ns at 300 K and 1 atm pressure (pressure relaxation time of 2.0 ps). Finally, production run was carried out under NPT ensemble for 20 ns. The binding energy for the hepcidin-GDP complex was

evaluated over last 2 ns (1000 frames) trajectory using Molecular Mechanics-Poisson Boltzmann Surface Area (MM-PBSA) method.[7]

Animal treatment:

Animals were divided into four groups (n=5/group) *i.e.* control, control+GDP+$FeSO_4$ group, LPS+$FeSO_4$ and LPS+$FeSO_4$+GDP group. To observe the effect of GDP+$FeSO_4$ on LPS induced inflammation state, initially mice were pre-treated with the $FeSO_4$ (2 mg/kg) and GDP (30 mg/kg) intraperitoneally for 30 minute , followed by LPS treatment (Sigma-Aldrich, USA 0.1 mg/kg[8-10] of body weight) intraperitoneally till 6 hour. The Mice were euthanized and tissues were harvested. Tissues were isolated and were stored at –80°C for further studies.

References

1. Case, D., *et al.* AMBER 11. (University of California, San Francisco, 2010).
2. Cornell, W.D., Cieplak, P., Bayly, C.I. & Kollmann, P.A. Application of RESP charges to calculate conformational energies, hydrogen bond energies, and free energies of solvation. *J. Am. Chem. Soc.* **115**, 9620-9631 (1993).
3. Wang, J., Wang, W., Kollman, P.A. & Case, D.A. Automatic atom type and bond type perception in molecular mechanical calculations. *J. Mol. Graph. Model.* **25**, 247-260 (2006).
4. Wang, J., Wolf, R.M., Caldwell, J.W., Kollman, P.A. & Case, D.A. Development and testing of a general amber force field. *J. Comput. Chem.* **25**, 1157-1174 (2004).
5. Jorgensen, W.L., Chandrasekhar, J., Madura, J.D., Impey, R.W. & Klein, M.L. Comparison of simple potential functions for simulating liquid water. *J. Chem. Phys.* **79**, 926-935 (1983).
6. Jorgensen, W.L., Maxwell, D.S. & Tirado-Rives, J. Development and testing of the OPLS all-atom force field on conformational energetics and properties of organic liquids. *J. Am. Chem. Soc.* **118**, 11225-11236 (1996).
7. Kollman, P.A., *et al.* Calculating structures and free energies of complex molecules: Combining molecular mechanics and continuum models. *Acc. Chem. Res.* **33**, 889-897 (2000).
8. Rizzardini, M., *et al.* Kupffer cell depletion partially prevents hepatic heme oxygenase 1 messenger RNA accumulation in systemic inflammation in mice: Role of interleukin 1β. *Hepatology* **27**, 703-710 (1998).
9. De Domenico, I., *et al.* Hepcidin mediates transcriptional changes that modulate acute cytokine-induced inflammatory responses in mice. *The Journal of clinical investigation* **120**, 2395-2405 (2010).
10. Fatih, N., *et al.* Natural and synthetic STAT3 inhibitors reduce hepcidin expression in differentiated mouse hepatocytes expressing the active phosphorylated STAT3 form. *J. Mol. Med.* **88**, 477-486 (2010).

LEGENDS OF SUPPLEMENTARY FIGURES:

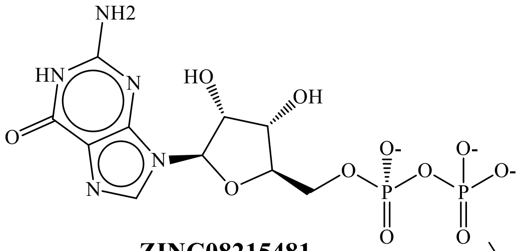

ZINC08215481

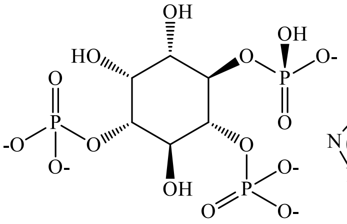

ZINC24433941

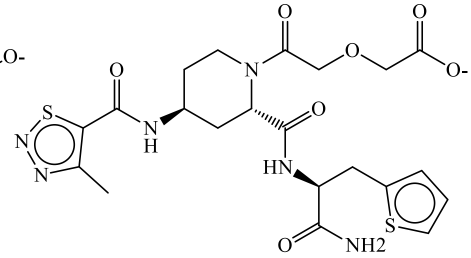

ZINC04221664

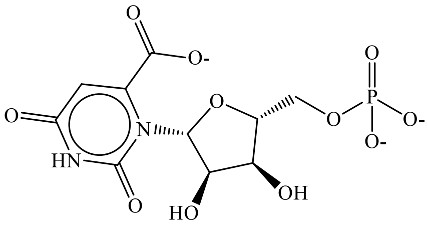

ZINC04095542

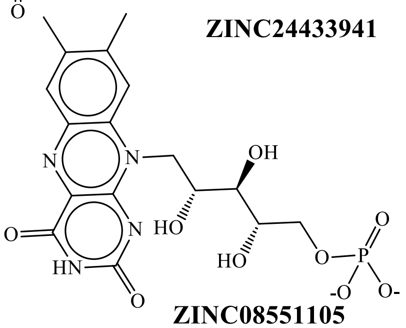

ZINC08551105

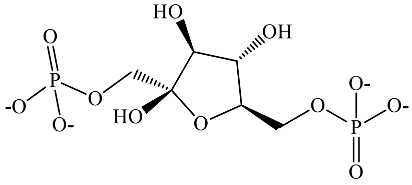

ZINC04096694

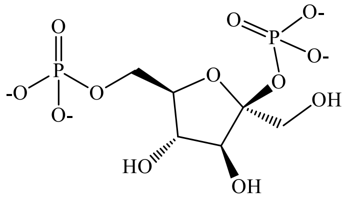

ZINC04228295

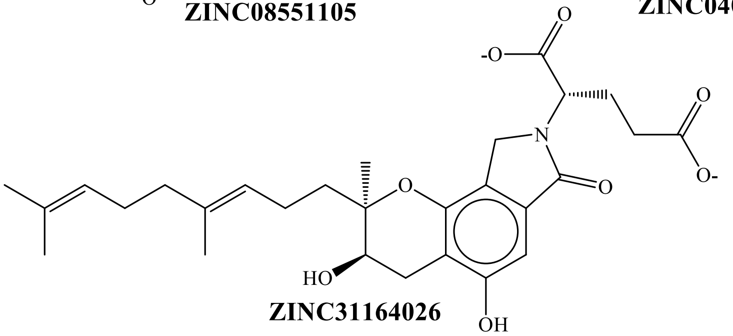

ZINC31164026

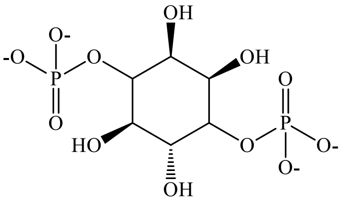

ZINC13527007

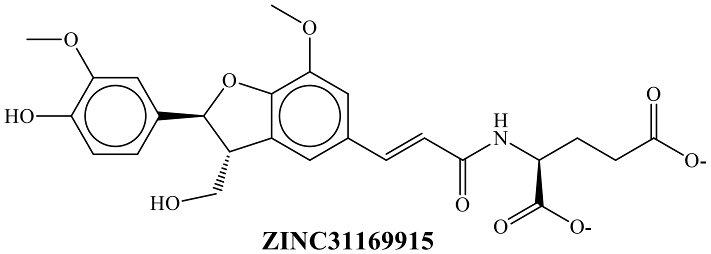

ZINC31169915

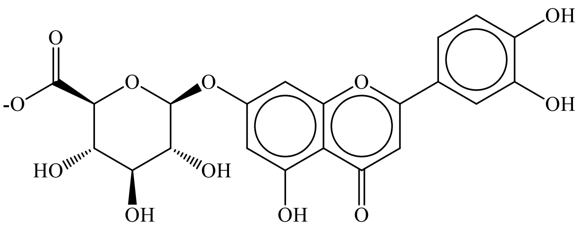

ZINC04654620

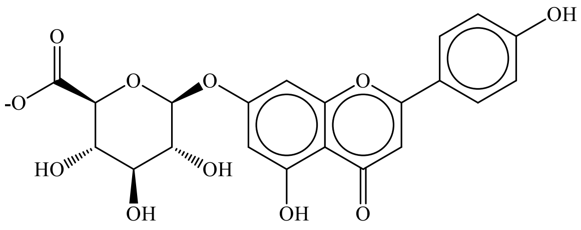

ZINC13543704

Figure S1. 2- Dimensional structures of selected 12 ligands. The ZINC IDs are provided in the figure. Chemical names for each ligand can be obtained from table 2.

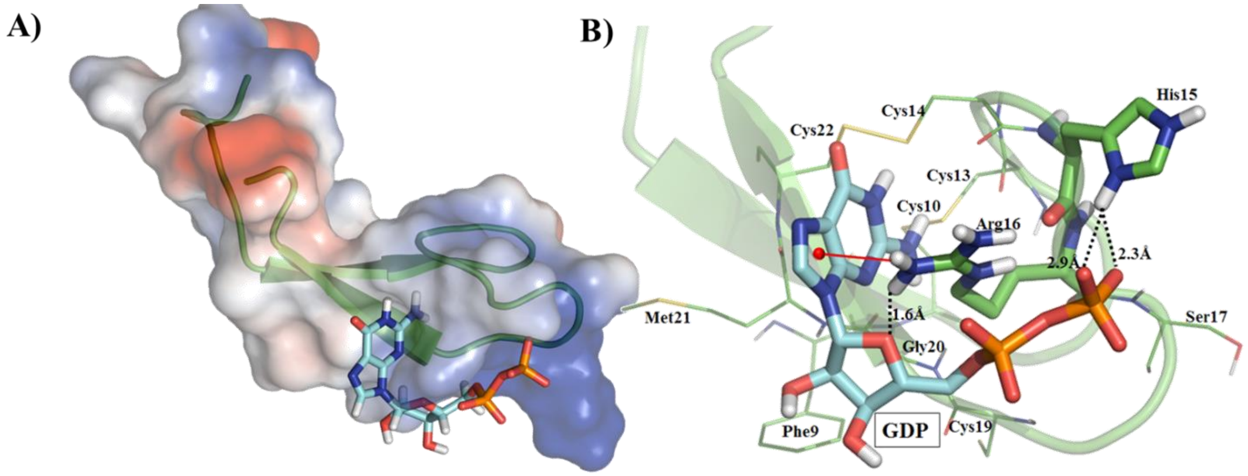

Figure S2. A) Electrostatic potential surface of hepcidin. The ligand binding region carries a highly electropositive potential and therefore accommodate the electronegative ligand moiety. For the same reason, aromatic rings present in the molecule also favour the ligand binding. B) Docked pose of GDB in the active site of hepcidin.

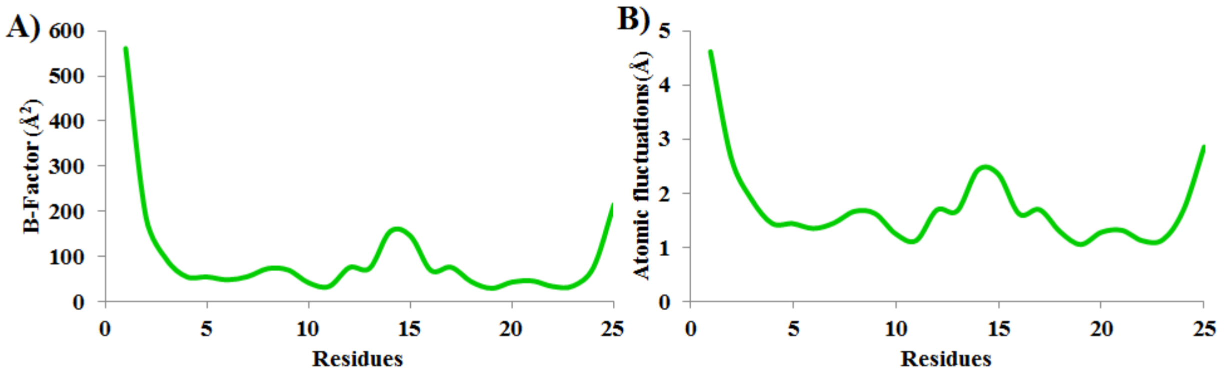

Figure S3. A) B-factor and B) atomic fluctuations for each residue in hepcidin.

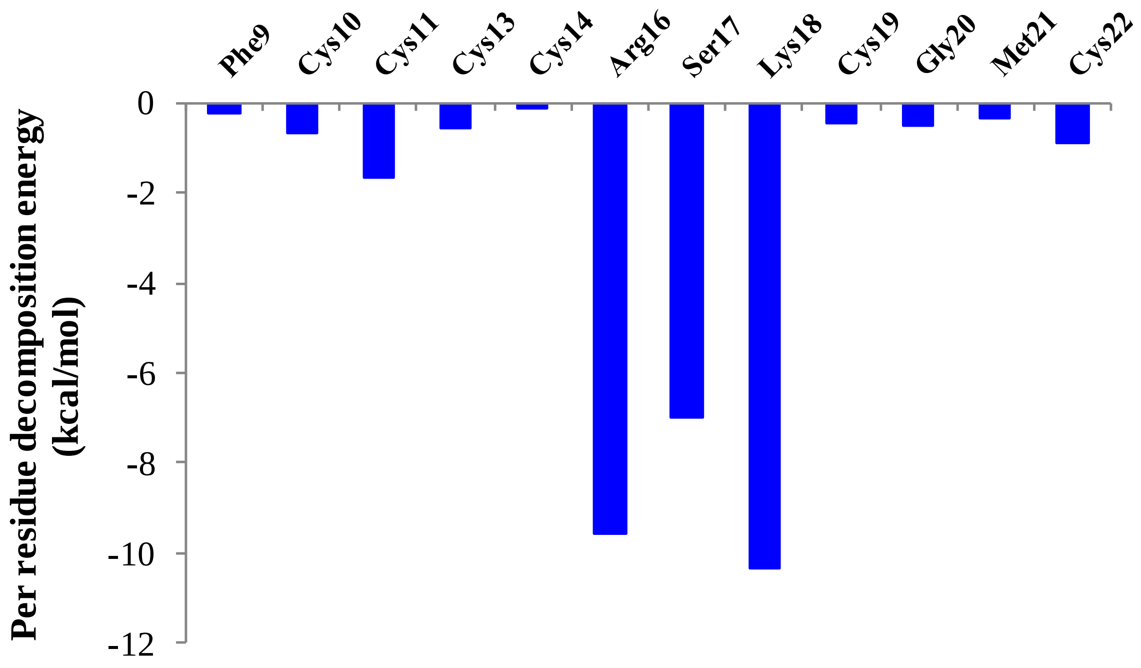

Figure S4. Per residue decomposition energy analysis for GDP-hepcidin complex.

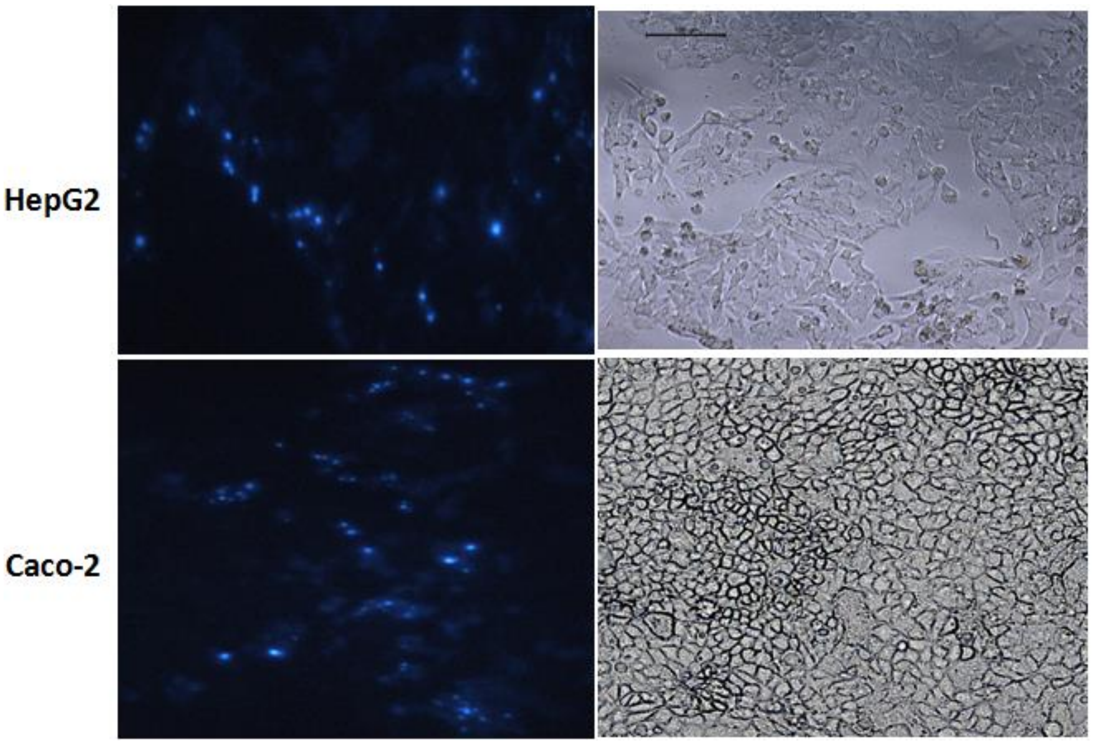

Figure S5: Internalization of MANT-GDP fluorescence signals were observed in HepG2 and Caco-2 cells.

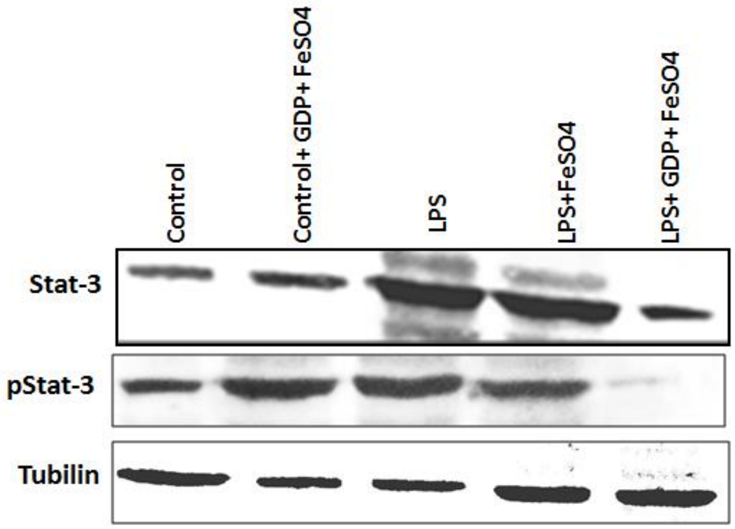

Figure S6: LPS induced Inflammation on treatment with GDP+$FeSO_4$ reduces phosphorylation of Stat-3 expression. Protein extract were prepared from liver and were immunoblotted with antibodies against total Stat-3, phospho-Stat-3. Tubilin was used as a loading control. The blots shown are representative of 3 independent experiments for each time point.

Table S1: Natural compound libraries used for screening of hepcidin binding agents.

	**Name of Library**	**No of Compounds**
1	AnalytiCon Discovery Natural Derivatives	25897
2	Indofine Natural Products	144
3	Nubbe Natural Products	588
4	TimTec Natural Derivatives	4943
5	UEFS Natural Products	473
6	Ambinter Natural Products	18679
7	Specs Natural Products	651
**Total**		**68,752**

Table S2: List of top 12 selected compounds exhibited highest binding affinity to hepcidin binding in preliminary virtual screening.

S. No.	ZINC Code	Compounds	Internal Energy	Grid Score	Molecular Weight
1	ZINC08215481	Guanosine diphosphate (GDP)	4.65	-47.58	440.17
2	ZINC24433941	1D-myo-inositol1,4,5-trisphosphate	17.52	-47.10	415.05
3	ZINC04221664	2-[[2-[[1-carbamoyl-2-(2-thienyl)ethyl]carbamoyl]-4-(4-methylthiadiazol-5-yl)carbonylamino-1-piperid	16.46	-46.93	537.6
4	ZINC04095542	Orotidine 5'-phosphate	11.55	-46.90	365.16
5	ZINC08551105	Riboflavin Monophosphate	17.52	-46.46	454.32
6	ZINC04096694	D-Fructofuranose1,6-bisphosphate	2.06	-45.31	336.08
7	ZINC04228295	D-Fructose 2,6-bisphosphate	6.39	-44.59	336.08
8	ZINC31164026	(S)-2-((2R,3R)-2-((E)-4,8-dimethylnona-3,7-dien-1-yl)-3,5-dihydroxy-2-methyl-7-oxo-3,4-dihydropyrano[2,3-e]isoindol-8(2H,7H,9H)-yl)pentanedioate	23.51	-44.59	513.57
9	ZINC13527007	1D-myo-inositol 1,4-bisphosphate	5.89	-44.56	336.08
10	ZINC31169915	(2S)-2-[[(E)-3-[(2S,3R)-2-(4-hydroxy-3-methoxy-phenyl)-3-(hydroxymethyl)-7-methoxy-2,3-dihydrobenzof	23.81	-44.45	499.46
11	ZINC04654620	Luteolin7-O-beta-D-glucosiduronate	9.73	-41.55	461.35
12	ZINC13543704	Apigenin-7-O-beta-D-glucuronide	16.61	-39.89	445.35

Table S3: Reported Ferroportin interacting residues of hepcidin in molecular docking studies.

Sl No.	Hepcidin Residue	Ferroportin Residue Interacting with Hepcidin
1	Phe9	Thr329
2	His15	Ile452
3	Arg16	Ser449<br>Pro451
4	Ser17	Ile452
5	Lys18	Ile341<br>Pro451
6	Cys19	Leu337<br>Pro451
7	Gly20	Glu448
8	Met21	Cys326<br>Thr329<br>Glu448
9	Cys22	Glu448

Table S4: List of primer for semi quantitative RT-PCR. The quantitative RT-PCR was performed using the following primers:

**Mouse target gene**	**Primer(5'- 3')**
m GAPDH-F	5'- GTGGAGATTGTTGCCATCAACGA-3'
m GAPDH-R	5'-CCCATTCTCGGCCTTGACTGT-3'
m Hamp-F	5'-GGCACTCAGCACTCGGACCCA-3'
m Hamp-R	5'-TTGGTATCGCAATGTCTGCCCTGC-3'
m DMT 1-F	5'-GGAAGTCATTGGCTCAGCCATCGC-3'
m DMT1-R	5'-AGTACTTGGCTCTGGCTGGGCTTC-3'
m TFR1-F	5'-GGGAGTCGCAAATGCCCTCTCTG-3'
m TFR1-R	5'-GTCCAACCCCGCACTAAAAGCTGC-3'
